# Data on Singapore longitudinal early development study (SG-LEADS)

**DOI:** 10.1016/j.dib.2024.110203

**Published:** 2024-02-17

**Authors:** Wei-Jun Jean Yeung, Xuejiao Chen

**Affiliations:** a The Agency for Science, Technology and Research (A*STAR), Singapore; b Yong Loo Lin School of Medicine and Centre of Family and Population Research, National University of Singapore. 30 Medical Drive, Brenner Center for Molecular Medicine, Singapore 117609; c Yong Loo Lin School of Medicine and Centre of Family and Population Research, National University of Singapore. MD1-12N, 10 Medical Dr, Singapore 117597

**Keywords:** Early childhood development, Cognitive, Social-psychological development, Health, Singapore, Home environment, Child care, Neighborhood

## Abstract

The Singapore Longitudinal Early Development Study (SG-LEADS) seeks to understand factors that can enhance or hinder Singaporean children's early childhood development with an aim to inform public policies that can help each child reach his or her potential. SG-LEADS is a nationally representative household survey that focuses on Singaporean households with children aged 0–6 at the baseline. It adopts a multi-stage probability sampling — clustered and stratified sampling strategy — with an oversample of the low-income households residing in 1–3-room HDB (Housing Development Board) public housing units. In-home face-to-face interviews were conducted on the computer-assisted personal interviewing (CAPI) systems. The baseline survey was conducted between 2018 and 2019. Up to two eligible children and their primary caregivers were interviewed in each household. In total, 5,005 Singaporean children aged 0–6 in 3,476 households and their primary caregivers were successfully interviewed. In 2021, 4,351 children in 3,017 households were successfully re-interviewed.

The contents of SG-LEADS are designed based on theories in multiple disciplines including sociology, psychology and economics about how multiple contexts (home, out-of-home care institution, community and state) interact to shape the multiple domains of child development. The survey includes assessments of children's motor, social-emotional well-being, language and cognitive skills, and biometric measures. Rich data about the family's socioeconomic, demographic and cultural backgrounds, family structure, family relations, home environment, social support, food security, financial distress, public program participation, and neighborhood characteristics and cohesion were collected. Comprehensive information was obtained about the target child's early childcare and preschool arrangements, children's time use (through time diaries), technology use, enhancement activities, nutrition intake and more. Primary caregivers’ social-emotional well-being, cognitive skills, and parenting behavior were also assessed. In Wave 2, a special module about the family's COVID-19 experiences and responses was added.

Descriptive statistics were presented on the study website. Multivariate analyses were conducted based on the SG-LEADS dataset with a variety of robust methodologies such as structural equation modeling, fixed effect analysis, lagged dependent variable model, hierarchical regression analyses, mediation analysis, propensity score matching, and so forth. Thus far, 14 journal articles have been published with a dozen or so papers under review. These analyses cover a wide range of topics, including but not limited to, 1) the impact of socioeconomic status on children's development such as infants' vocabulary, children's academic achievement and socio-emotional development, 2) the impact of paternity leave-taking on child outcome (3) how food insecurity affects children's behavior, (4) family experiences and resilience during COVID-19, (6) childcare arrangements and children's behavior and cognitive development, (6) children's ability to delay gratification in early childhood, (7) children in cross-national families, 8) children's time use, (9) family and community social capital and child development.

The rich data in this longitudinal study provide many opportunities to research a wide range of topics related to early child development in an Asian context. This dataset holds tremendous potential to uncover valuable insights and inform evidence-based policy interventions to support optimal early childhood development. International comparative studies can also be conducted with similar surveys conducted in other countries.

Specifications Table

**Table:** 

Subject	Sociology, Developmental and Educational Psychology, Social Sciences
Specific subject area	Early childhood, Family, education, developmental psychology.
Type of data	The raw data is in table, with each respondent being one row, and each column representing one variable.
Data collection	The data was collected through in-home face-to-face interviews in the Computer-Assisted Personal Interview (CAPI) system on Samsung Tablets. For each interview, one interviewer interviewed the child's primary caregiver, and another interviewer interviewed the target child. The Primary caregiver of the target child was the main respondent while in the child outcome assessments, interviewers interact directly with the child. The main questionnaire set includes a household information form, household booklet, child booklet, and child assessment. The questionnaires and data can be accessed online (https://fass.nus.edu.sg/cfpr/sgleads/documentation/). The origins of the questions and scales in the questionnaire can be found in the cross-wave questionnaire constructs (https://fass.nus.edu.sg/cfpr/wp-content/uploads/sites/17/2023/08/Cross-Wave-Questionnaire-Constructs.pdf).
Data source location	• Institution: National University of Singapore • City: Singapore • Country: Singapore • 1.3521° N, 103.8198° E
Data accessibility	Repository name: NUS Scholar Bank Data identification number: https://doi.org/10.25540/DNBB-GFD0 Direct URL to data: https://scholarbank.nus.edu.sg/handle/10635/244540 Instructions for accessing these data: users need to fill in a short request form before being allowed to download all the data material.
Related research article	Yeung, W.J. and Chen, X. (2023) Achievement Gaps before School in Singapore: Family SES, Parental Values and Young Children's Self-Regulation. Early Childhood Research Quarterly, 65, 352-362. https://doi.org/10.1016/j.ecresq.2023.07.012

## 1 Value of the Data

• The SG-LEADS dataset is a nationally representative longitudinal dataset, with an extensive range of variables including information on the household members, the primary caregiver's values and thoughts, family process, home environment, parental financial and time investment in children, and children's time use. The information spans multiple contexts including home, schools, community and the state.
• The dataset would be a valuable resource for researchers, practitioners, and policymakers interested in early childhood development. It would enable them to conduct in-depth research, design effective interventions, and develop evidence-based policies that can positively impact the lives of children and contribute to their overall well-being.
• The dataset could be used to perform correlation and predictive analysis of factors that contributed to children's developmental outcomes and track children's development over time.
• The study is comparable with other international studies such as the Child Development Supplement of the Panel Study of Income Dynamics (CDS of PSID) and National Longitudinal Survey of Youth (NLSY), Early Childhood Longitudinal Study (ECLS), and Growing Up in Australia (LSAC) which allows a comparative analysis across different socioeconomic contexts, highlighting areas for targeted interventions.

## 2 Background

The Singapore Longitudinal Early Development Study (SG-LEADS) used innovative methods to understand the factors that can promote Singapore children's healthy development. The study was conducted at the National University of Singapore Center for Family and Population Research. Broadly, the main research questions examined are how family, daycare/school, community, and state interact to shape the human development of Singapore children, and how these investments will affect social mobility in Singapore. The survey focuses on development in early childhood. Special attention has been paid to disadvantaged sub-groups. In the context of globalized economies and rising income inequality, a major challenge in many countries including Singapore is how to raise the next generation to be as educated and productive as possible. One of the biggest concerns of Singapore policymakers is the possibility of second or third-generation poverty, i.e. a cycle of poverty. Specifically, the concern is that some youth from currently disadvantaged families will do poorly in school and integrate poorly into Singapore's increasing ‘high-tech’ and diversified labor market. This study can offer valuable insights into the myriad factors that shape children's and youth's development across physical, socio-emotional, and cognitive domains and sheds light on potential policy interventions to mitigate the impacts of these factors.

## 3 Data description

The SG-LEADS data team has already transformed some data files for users which includes creating constructed variables, cleaning data, and removing confidential information. The main dataset of each wave includes the data in the household Information Form (or Screener), Household Booklet, Child Booklet, and Child Assessment. The Household Information Form contains the demographic information of all members. The Household booklet includes information on the neighborhood, the primary caregiver's values and thoughts, family process, home environment and the family's economic information. The Child Booklet of each child includes information on the child's health, home environment, language, psychological wellbeing, school, parenting, child care arrangement, time diary and information about absent parents. The Child Assessment includes children's assessment results in achievement tests, working memory and delay of gratification. Table 1 lists the dataset and related files in each wave:

**Table 1:** Types of data files.

Data file name	Contents	Data structure
1. Wave 1 questionnaires	The questionnaire set of each wave includes 4 booklets: household information form, household booklet, child booklet.	organized by wave separately
2. Wave 1 data	Combines all four booklets—the Household Information Form, Household Booklet, Child Booklet, and Child Assessment of wave 1	Data is organized at the child level. Each record contains information for a target child in each household
3. Wave 1 study guide	Study guide of wave 1	organized by wave separately
4. Wave 1 technical reports	12 technical reports on topics such as response rate, sampling weights, validity of constructs.	organized by wave separately
5. Wave 2 questionnaires	The questionnaire set of each wave includes 4 booklets: household information form, household booklet, child booklet.	organized by wave separately
6. Wave 2 data	Combines all four booklets—the Household Information Form, Household Booklet, Child Booklet, and Child Assessment of wave 2	Data is organized at the child level. Each record contains information for a target child in each household
7. Wave 2 study guide	Study guide of wave 2	organized by wave separately
8. Wave 2 technical reports	11 technical reports on topics such as response rate, sampling weights, validity of constructs.	organized by wave separately

## 4 Experimental design, materials and methods

### 4.1 Sample design

The wave 1 survey is based on a nationally representative sample of resident households with at least one child below 7 years of age. The sampling frame consists of 6,575 addresses. In selecting the sample, we adopted a multi-stage probability sampling - clustered and stratified sampling strategy. The sampling frame is stratified by three broad public housing types, i.e., HDB 1–3 room flats, HDB 4-room flats, and other remaining housing types (including 5-room HDB, executive HDB, private condominiums, landed properties, and others). We drew the dwelling units so that 40% of the sample was from HDB 1–3 room flats, 30% was from HDB 4-room flats, and 30% was from the remaining housing types. The study oversamples the population residing in 1–3-room HDB units as a proxy for low-income households. The sampling unit here is the dwelling unit.

In the second stage, within each broad housing type, we included all 55 planning areas (PAs) within the 5 URA regions in Singapore (Central, East, North, North-East, West) with residents under 7 years of age. The required number of addresses in each PA was calculated proportionately to the number of households with at least one child under 7 years old (PPS). Within each PA, the addresses were randomly selected. These produced dwelling units of different sizes (a proxy for SES) in 55 PAs across the 5 regions in Singapore.

In the third stage, we randomly selected up to 2 eligible children aged under 7 years old in each selected household. In-home face-to-face interviews were conducted on the computer-assisted personal interviewing (CAPI) systems with the selected child(ren) and the primary caregiver. The baseline survey (wave 1) was conducted between 2018 and 2019. In total, 5,005 Singaporean children aged 0–6 in 3,476 households and their primary caregivers were successfully interviewed.

In 2021 (wave 2), the same children and their primary caregivers were followed up with 4,351 children in 3,017 households being successfully re-interviewed.

In addition, three subprojects based on sub-samples of the SG-LEADS national sample were conducted: (1) a laboratory experiment of early childhood language skills development, (2) an intervention program to improve children's social skills, and (3) an ethnographic study on children growing up in cross-cultural families.

### 4.2 Interviews with children and primary caregivers

For each interview, one interviewer interviewed the child's primary caregiver, and another interviewer interviewed the target child.

Most of the questionnaires were administered with the primary caregiver Interviewer reading the questions and options aloud to the respondent. For those questions, the tablet is positioned in a way that the primary caregiver can see the screen clearly while the Interviewer is reading the questions. For self-administered questions, the primary caregiver interviewer handed the tablet to the primary caregiver and let the primary caregiver work on the questions on his/her own unless there were clarifications.

Meanwhile, the child Interviewer administered the Child Assessment component to the child. For children 3 years old and above, the Child Assessment component included an academic achievement test (Woodcock-Johnson Test of Achievement IV) in math and reading, a Delay of Gratification test, a Digit Span task to measure working memory, as well as height and weight measurements. For children below 3 years old, the Child Assessment component only included a height and weight measure.

In administering the Child Assessment component, the child interviewers were instructed to ask the primary caregiver for an area in the house conducive to the assessments. Having a room separate from the primary caregiver interview is ideal. In cases where there was no separate room, the Interviewers should sit as far as practicable from each other to avoid distractions caused by hearing what is happening in the other interview. For children who are shy and do not want to be separated from the primary caregiver, the primary caregiver can stay nearer the child before or during the assessment (without intervening in the assessment).

Additionally, child interviewers were required to respond to an interviewer observation form at the end of the interview. Interviewers reported their observation of the physical home environment, and the interaction between the primary caregiver and each target child separately. The observation was only reported by one interviewer.

### 4.3 Data cleaning

After data collection, a preliminary examination was performed to check the number of missing values and data consistency. Open questions were recorded.

### 4.4 Questionnaires

The main questionnaire set includes a household information form, household booklet, child booklet, and child assessment. The questions are mainly derived from the questionnaires of Child Development Supplement (CDS) of Panel Study of Income Dynamics (PSID) 1997 to 2014. SG-LEADS questionnaires can be accessed online (https://fass.nus.edu.sg/cfpr/sgleads/documentation/).

Refer to the cross-wave questionnaire construct document of each wave for the source of each question (https://fass.nus.edu.sg/cfpr/wp-content/uploads/sites/17/2023/08/Cross-Wave-Questionnaire-Constructs.pdf).

## Limitations

A limitation of this survey is the lack of biomarker data collection from the children and their primary caregiver.

## Ethics Statement

Written approval has been granted for the SG-LEADS study by the National University of Singapore Institutional Review Board: IRB #: LS-17-326, title: Building Human Capacity in Singapore's Population: Testing Innovations in Human Development – Panel Survey Component [Simplified Title: Singapore Longitudinal Early Development Study (SG-LEADS) - Panel Survey Component].

Written informed consent was obtained from the primary caregivers of each participating household, both for themselves and on behalf of their children.

## CRediT authorship contribution statement

**Wei-Jun Jean Yeung:** Funding acquisition, Conceptualization, Data curation, Supervision, Writing – review & editing. **Xuejiao Chen:** Data curation, Writing – original draft.

## Data Availability

Singapore Longitudinal Early Development Study (SG LEADS) (Original data) (NUS Scholar Bank).

